# Supplementary material for: Machine Learning Accelerates Crystallization for Structure Determination
Source: Angew Chem Int Ed Engl. 2026 May 4;65(25):e1218503. doi: 10.1002/anie.1218503 (PMC13266958; doi:10.1002/anie.1218503)

checkCIF/PLATON report

Structure factors have been supplied for datablock(s) lcz076

THIS REPORT IS FOR GUIDANCE ONLY. IF USED AS PART OF A REVIEW PROCEDURE FOR PUBLICATION, IT SHOULD NOT REPLACE THE EXPERTISE OF AN EXPERIENCED CRYSTALLOGRAPHIC REFEREE.

No syntax errors found.   CIF dictionary   Interpreting this report

Datablock: lcz076

Bond precision: C-C = 0.0093 A   Wavelength=1.54184

Cell:	a=8.6927(1)	b=12.2745(2)	c=18.6304(2)
	alpha=102.528(1)	beta=95.484(1)	gamma=103.606(1)
Temperature:	100 K		

	Calculated	Reported
Volume	1863.40(4)	1863.40(4)
Space group	P -1	P -1
Hall group	-P 1	-P 1
Moiety formula	C23 H12 Ag3 F18 N7 O, C8 H9 N O	C15 H3 Ag3 F18 N6, 2(C8 H9 N O)
Sum formula	C31 H21 Ag3 F18 N8 O2	C31 H21 Ag3 F18 N8 O2
Mr	1203.17	1203.17
Dx,g cm-3	2.144	2.144
Z	2	2
Mu (mm-1)	13.777	13.777
F000	1164.0	1164.0
F000'	1169.40	
h,k,lmax	11,15,23	10,15,23
Nref	7948	7548
Tmin,Tmax	0.092,0.252	0.453,1.000
Tmin'	0.006	

Correction method= # Reported T Limits: Tmin=0.453 Tmax=1.000
AbsCorr = MULTI-SCAN

```
Data completeness= 0.950           Theta(max)= 77.919

R(reflections)= 0.0506( 6473)                            wR2(reflections)=
                                                         0.1337( 7548)
S = 1.079                 Npar= 591
```

The following ALERTS were generated. Each ALERT has the format
test-name_ALERT_alert-type_alert-level.
Click on the hyperlinks for more details of the test.

Alert level C

```
PLAT042_ALERT_1_C Calc. and Reported MoietyFormula Strings  Differ     Please Check
              Calc: C23 H12 Ag3 F18 N7 O, C8 H9 N O
              Rep.: C15 H3 Ag3 F18 N6, 2(C8 H9 N O)
PLAT213_ALERT_2_C Atom F4              has ADP max/min Ratio .....        3.9 prolat
PLAT213_ALERT_2_C Atom F2A             has ADP max/min Ratio .....        3.3 prolat
PLAT213_ALERT_2_C Atom F4A             has ADP max/min Ratio .....        3.3 oblate
PLAT342_ALERT_3_C Low Bond Precision on  C-C Bonds ..............    0.00932 Ang.
PLAT420_ALERT_2_C D-H Bond Without Acceptor  N00R     --H00B     .     Please Check
PLAT906_ALERT_3_C Large K Value in the Analysis of Variance ......      5.230 Check
PLAT911_ALERT_3_C Missing FCF Refl Between Thmin & STh/L=    0.600         38 Report
               -9  4  0,  -6  6  0,  -9 -3  1,   9  3  1,   9 -4  2, -10  7  2,
                9 -6  3, -10  5  3, -10  6  3, -10  3  4, -10  4  4, -10  5  4,
                9 -7  5,  -8 -4  5, -10  2  5, -10  3  5,  -9  3  5, -10  4  5,
              -10  5  5, -10  2  6, -10  3  6, -10  4  6, -10  5  6, -10  1  7,
              -10  2  7, -10  3  7, -10  4  7, -10  2  8, -10  3  8,   7-10  9,
                   (    8 More Missing: see the .ckf listing file)
PLAT934_ALERT_3_C Number of (Iobs-Icalc)/Sigma(W) > 10 Outliers ..          1 Check
                7-10  7,
PLAT977_ALERT_2_C Check Negative Difference Density on H00B      .      -0.42 eA-3
PLAT977_ALERT_2_C Check Negative Difference Density on H00C      .      -0.36 eA-3
PLAT977_ALERT_2_C Check Negative Difference Density on H015      .      -0.35 eA-3
```

Alert level G

```
PLAT002_ALERT_2_G Number of Distance or Angle Restraints on AtSite          7 Note
PLAT003_ALERT_2_G Number of Uiso or U(i,j)  Restrained non-H-Atoms          3 Report
PLAT007_ALERT_5_G Number of Unrefined Donor-H Atoms .............           4 Report
              H00A  H00B  H00C  H00D
PLAT083_ALERT_2_G SHELXL Second Parameter in WGHT  Unusually Large      11.60 Why ?
PLAT154_ALERT_1_G The s.u.'s on the Cell Angles are Equal ..(Note)      0.001 Degree
PLAT176_ALERT_4_G The CIF-Embedded .res File Contains SADI Records          3 Report
PLAT186_ALERT_4_G The CIF-Embedded .res File Contains ISOR Records          1 Report
PLAT242_ALERT_2_G Low    'MainMol' Ueq as Compared to Neighbors of         C3 Check
PLAT242_ALERT_2_G Low    'MainMol' Ueq as Compared to Neighbors of       C00Z Check
PLAT242_ALERT_2_G Low    'MainMol' Ueq as Compared to Neighbors of       C01A Check
PLAT242_ALERT_2_G Low    'MainMol' Ueq as Compared to Neighbors of       C01B Check
PLAT242_ALERT_2_G Low    'MainMol' Ueq as Compared to Neighbors of       C01C Check
PLAT301_ALERT_3_G Main Residue  Disorder .............(Resd    1)          6% Note
PLAT432_ALERT_2_G Short Inter X...Y Contact  F1A      ..C01N     .       2.96 Ang.
                                                      x,y,z  =      1_555 Check
PLAT434_ALERT_2_G Short Inter HL..HL Contact F00C     ..F2A      .       2.84 Ang.
                                                -1+x,-1+y,z  =      1_445 Check
```

```
PLAT720_ALERT_4_G Number of Unusual/Non-Standard Labels .........         79 Note
              Ag01    Ag02    Ag03    F004    F005    F006    F007    F008
              F009    F00A    F00B    F00C    F00D    F00E    F00F    F00G
              O00H    F00I    N00J    N00K    F00L    N00M    N00N    O00O
              N00P    N00Q    N00R    H00A    H00B    N00T    H00C    H00D
              C00V    C00W    H00W    C00X    C00Y    H00Y    C00Z    C010
              H010    C011    C012    H012    C013    C014    C015    H015
              C016    C017    H017    C018    C019    C01A    C01B    C01C
              C01D    H01A    H01B    H01C    C01E    H01D    H01E    H01F
              C01F    C01G    H01G    C01H    H01H    C01I    C01J    C01K
              H01K    C01L    H01L    C01M    C01N    C01Q    H01Q
PLAT860_ALERT_3_G Number of Least-Squares Restraints ............         21 Note
PLAT883_ALERT_1_G Absent Datum for _atom_sites_solution_primary ..     Please Do !
PLAT912_ALERT_4_G Missing # of FCF Reflections Above STh/L=  0.600        356 Note
PLAT933_ALERT_2_G Number of HKL-OMIT Records in Embedded .res File         10 Note
                9 -7  5,   9 -6  3, -10  5  3, -10  6  3,   9 -4  2, -10  7  2,
              -10  5  4, -10  8  3,  -9  4  0, -10  8  1,
PLAT941_ALERT_3_G Average HKL Measurement Multiplicity ..........         2.3 Low
PLAT969_ALERT_5_G The 'Henn et al.' R-Factor-gap value ..........      2.239 Note
              Predicted wR2: Based on SigI**2  5.97 or SHELX Weight 12.39
PLAT978_ALERT_2_G Number C-C Bonds with Positive Residual Density.          0 Info
```

```
   0 ALERT level A = Most likely a serious problem - resolve or explain
   0 ALERT level B = A potentially serious problem, consider carefully
  12 ALERT level C = Check. Ensure it is not caused by an omission or oversight
  23 ALERT level G = General information/check it is not something unexpected

   3 ALERT type 1 CIF construction/syntax error, inconsistent or missing data
  19 ALERT type 2 Indicator that the structure model may be wrong or deficient
   7 ALERT type 3 Indicator that the structure quality may be low
   4 ALERT type 4 Improvement, methodology, query or suggestion
   2 ALERT type 5 Informative message, check
```

Validation response form

Please find below a validation response form (VRF) that can be filled in and pasted into your CIF.

```
# start Validation Reply Form
_vrf_PLAT042_lcz076
;
PROBLEM: Calc. and Reported MoietyFormula Strings  Differ     Please Check
RESPONSE: ...
;
_vrf_PLAT213_lcz076
;
PROBLEM: Atom F4              has ADP max/min Ratio .....        3.9 prolat
RESPONSE: ...
;
_vrf_PLAT342_lcz076
;
PROBLEM: Low Bond Precision on  C-C Bonds ..............    0.00932 Ang.
RESPONSE: ...
;
_vrf_PLAT420_lcz076
;
```

```
PROBLEM: D-H Bond Without Acceptor  N00R     --H00B     .     Please Check
RESPONSE: ...
;
_vrf_PLAT906_lcz076
;
PROBLEM: Large K Value in the Analysis of Variance ......      5.230 Check
RESPONSE: ...
;
_vrf_PLAT911_lcz076
;
PROBLEM: Missing FCF Refl Between Thmin & STh/L=    0.600         38 Report
RESPONSE: ...
;
_vrf_PLAT934_lcz076
;
PROBLEM: Number of (Iobs-Icalc)/Sigma(W) > 10 Outliers ..          1 Check
RESPONSE: ...
;
_vrf_PLAT977_lcz076
;
PROBLEM: Check Negative Difference Density on H00B      .      -0.42 eA-3
RESPONSE: ...
;
# end Validation Reply Form
```

It is advisable to attempt to resolve as many as possible of the alerts in all categories. Often the minor alerts point to easily fixed oversights, errors and omissions in your CIF or refinement strategy, so attention to these fine details can be worthwhile. It is up to the individual to critically assess their own results and, if necessary, seek expert advice.

PLATON version of 17/09/2025; check.def file version of 17/09/2025

---

duplicate check

No duplication found

Datablock lcz076 - ellipsoid plot

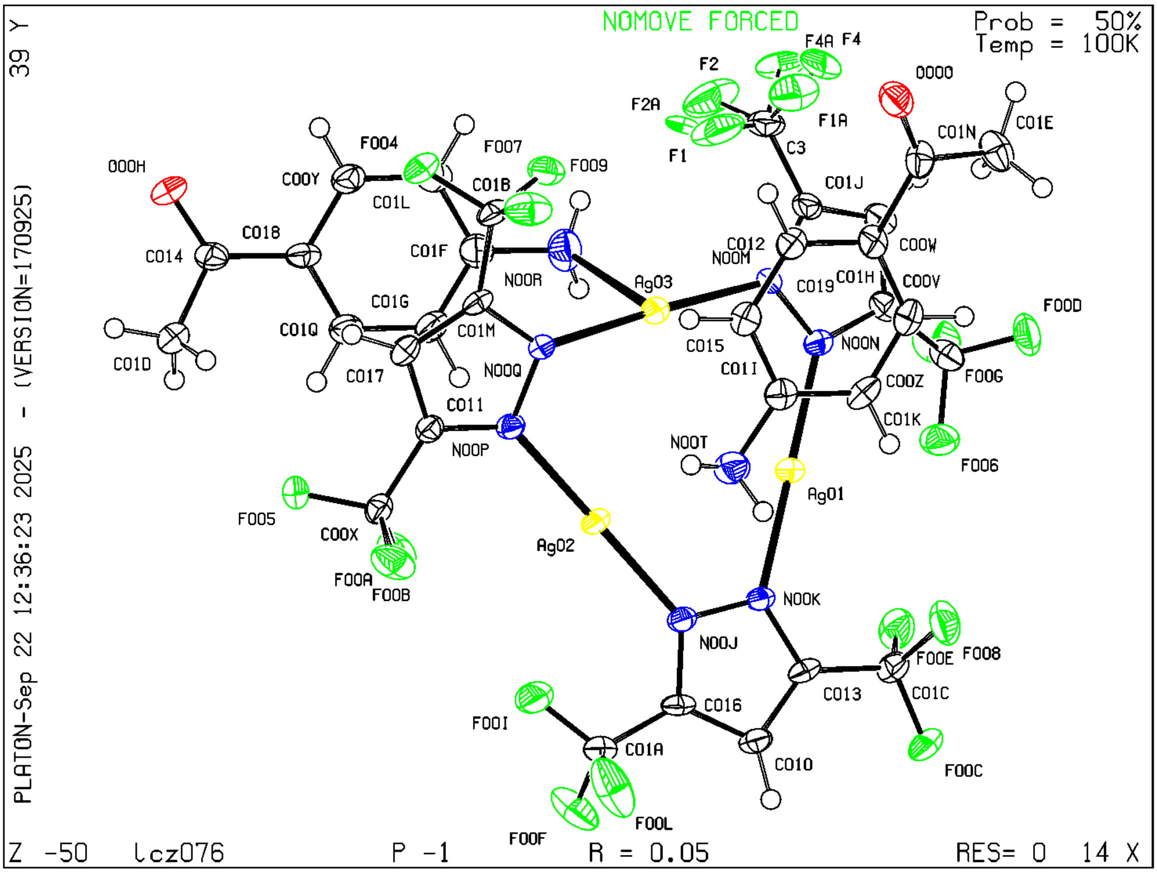

Supplement: Supplementary file 2 — Supporting File 2: anie72462‐sup‐0002‐DataFile.zip. [file ANIE-65-e1218503-s002.zip › supplementary_materials_crystallographic_data submit.zip!/cif_and_checkcif(1-114)/Ag3Pz3_76/Ag3Pz3_76_checkcif.pdf]
